# Cyp4a12-mediated retinol metabolism in stellate cells is the antihepatic fibrosis mechanism of the Chinese medicine Fuzheng Huayu recipe

**DOI:** 10.1186/s13020-023-00754-4

**Published:** 2023-05-09

**Authors:** Da-bing Ping, Xin Sun, Yuan Peng, Cheng-hai Liu

**Affiliations:** 1 Institute of Liver Diseases, Shuguang Hospital affiliated with Shanghai University of Traditional Chinese Medicine, 528 Zhangheng Road, Shanghai, 201203 China; 2 Shanghai Key Laboratory of Traditional Chinese Clinical Medicine, 528 Zhangheng Road, Shanghai, 201203 China; 3 Key Laboratory of Liver and Kidney Diseases, Ministry of Education, 528 Zhangheng Road, Shanghai, 201203 China

**Keywords:** Retinol metabolism, Cyp4a, Rae-1, NK cells, Liver fibrosis, FZHY

## Abstract

**Background:**

Hepatic stellate cells (HSCs), which contain multiple retinol-containing lipid droplets, are important profibrotic cells in liver fibrosis. Under Cyp4a12a/b oxidation, HSC activation was accompanied by the downregulation of genes involved in retinol metabolism, inducing RAE-1 production. By eliminating activated HSCs, NK cells expressing the activating receptor NKG2D are recruited to alleviate fibrosis. FZHY was found to significantly reduce the severity of liver fibrosis by inhibiting the activation and proliferation of HSCs. The molecular processes that govern retinol metabolism, on the other hand, are largely unexplored. This study focused on the regulation of Cyp4a12a/b by FZHY to elucidate the antifibrotic molecular mechanisms underlying the effect of FZHY on retinol metabolism.

**Methods:**

To investigate mechanisms and altered pathways of FZHY against carbon tetrachloride (CCl4)-induced liver fibrosis based on transcriptomics data. Bioinformatics analysis was used to screen its pharmacological targets. The predicted targets were confirmed by a series of in vitro and in vivo experiments, including mass spectrometry, in situ hybridization, immunofluorescence assays and real-time PCR. Then, the results were further characterized by recombinant adenovirus vectors that were constructed and transfected into the cultured primary HSCs.

**Results:**

Transcriptomics revealed that Cyp4a12a/b is nearly completely lost in liver fibrosis, and these effects might be partially reversed by FZHY therapy recovery. In vitro and in vivo studies indicated that Cyp4a12a/b deletion disrupted retinol metabolism and lowered Rae-1 expression. Activated HSCs successfully escape recognition and elimination by natural killer (NK) cells as a result of reduced Rae-1. Notablely, we discovered that FZHY may restore the Cyp4a12a/b capability, allowing the recovery of the cytotoxic function of NK cells against HSCs, and thereby reducing hepatic fibrosis by suppressing HSC activation.

**Conclusion:**

Our findings revealed a new role for Cyp4a in retinol metabolism in the development of hepatic fibrosis, and they highlight Cyp4a12/Rae-1 signals as possible therapeutic targets for antifibrotic medicines.

**Supplementary Information:**

The online version contains supplementary material available at 10.1186/s13020-023-00754-4.

## Introduction

Liver fibrosis represents an adaptive response to repeated chronic liver injury induced by various aetiologies, including viruses, alcohol consumption, or obesity [1]. Hepatic stellate cells(HSCs) are the characteristic cellular basis during the development of liver fibrosis. External injurious stimuli will gradually induce quiescent HSCs to develop a proliferative myofibroblastic phenotype. Activated HSCs synthesize extracellular matrix and secrete metalloproteinases, leading to disturbance of the liver microenvironment [2]. Therefore, it is crucial to understand the role of HSCs in recovery from liver fibrosis.

Recently, the transformation of lipid droplets in HSCs has been extensively reported [3, 4]. In healthy individuals, 80–90% of retinoids are stored in quiescent HSCs. Upon suffering from liver injuries, HSCs gradually lose their lipid droplets and display an activated phenotype, and restoration of lipid droplet accumulation inhibits HSC activation [5]. Triglycerides, retinyl esters and cholesterol esters in lipid droplets are necessary to maintain the normal physiological function of HSCs [6]. Retinol in HSCs can be oxidized into retinaldehyde by alcohol dehydrogenases (ADHs), which is further oxidized and metabolized to retinoic acid by retinaldehyde dehydrogenase (Raldh). Then, retinoic acid binds to nuclear receptors such as retinoic acid receptors (RARs) and retinoid X receptors(RXRs), which regulate target gene transcription by binding to retinoic acid-responsive elements in DNA. Retinoic acid early induction gene 1 (Rae-1), a ligand for NKG2D, has been identified to activates NK cells through ligand receptor binding [7]. However, the metabolic transition of retinol in HSCs is still unclear. Therefore, elucidating the mechanism of retinoic acid metabolism is important for ameliorating liver fibrosis.

Cytochrome P450 (CYP) is a gene superfamily composed of a large and diverse haem protein involved in the metabolism of a variety of xenobiotics and endogenous substrates. Among the genes of the CYP4A subfamily are microsomal omega-hydroxylase enzymes commonly used to metabolize fatty acids, eicosanoids and vitamin D [8]. To date, 4A11 and 4A22 from humans, 4a1, 4a2, 4a3, and 4a8 from rats and 4a10, 4a12a, 4a12b, and 4a14 from mice have been identified, and CYP4a12 is predominantly expressed in the livers of male mice. However, whether CYP4As participate in retinoic acid metabolism is unclear, although there is evidence that multiple cytochrome P450 genes, including CYP26, and CYP2C may play a role in RA oxidation [9, 10]. Therefore, exploring the effect of CYP on retinoic acid metabolism is urgently needed. In addition, Fuzheng Huayu Recipe (FZHY), a traditional Chinese medicine botanical preparation, has shown significant anti-hepatic fibrosis activity [11, 12]. Previous studies have had confirmed that FZHY can inhibit HSC activation, however, the specific targets and mechanisms of FZHY have not been elucidated [13]. Therefore, whether cytochrome P450-mediated retinoic acid metabolism contributes to the alleviation liver fibrosis by FZHY deserves further study.

In this study, we identified the regulation of retinoic acid metabolism by Cyp4a12a/b and further demonstrated the effect of retinoic acid-mediated changes in Rae-1 on NK cell killing sensitivity. Interestingly, FZHY promoted Rae-1 expression by restoring Cyp4a12a/b for more pronounced recognition from NK cells. Overall, our study confirms the important role of Cyp4a12a/b in retinoic acid metabolism and provides a rationale for FZHY as a pharmacological target for alleviating liver fibrosis.

## Materials and methods

### Ethics

All experimental procedures complied with the Guidelines for Experimentation of Shuguang Hospital, affiliated with Shanghai University of Traditional Chinese Medicine. The protocols were reviewed and approved by the Ethics Committee of the institution.

### Drug preparation and identification

The FZHY recipe consists of six crude herbs with a 1-day dose for adults: 8.0 g of Radix Salviae Miltiorrhizae, 4.0 g of Fermentation Mycelium Powder, 2.0 g of Fructus Schisandrae Chinensis, 2.0 g of Semen Persicae, 2.0 g of Pollen Pini, and 6.0 g of Gynostemma Pentaphyllammak. The FZHY powder was purchased and major ingredients were determined by Shanghai Sundise Medicine Technology Development Co. Ltd. for quality control as previously described [14] (Additional file 1: Figure S1).

### Mice

Male C57BL/6 mice weighing 25 ± 1 g were supplied by Beijing Vital River Laboratory Animal Technology. The mice were fed in the Experimental Animal Center of Shanghai University of Traditional Chinese Medicine (Licence No: SCXK[Hu]2020-0009) and were acclimatized to the animal centre conditions for 7 days before the experiments. The mice were given rodent laboratory chow and water ad libitum and maintained under controlled conditions with a temperature of 22–25 °C, relative humidity of 46–52%, and a 12/12-h light/dark cycle (lights on at 7:00 am). Our experiments were conducted in accordance with the Animal Ethics Committee of Shanghai University of Traditional Chinese Medicine (Licence No: SZY201804008).

### Carbon tetrachloride (CCl _4_ ) inductionof liver fibrosis

For liver fibrosis, mice were intraperitoneally injected with 10% CCl4/olive oil 2 ml/kg body weight three times a week, for 6 weeks [15]. Following pentobarbital sodium anaesthesia, all mice were sacrificed 24 h after the last injection. Serum and liver samples were harvested. For histology, tissue specimens were fixed in buffered formalin and embedded in paraffin wax (Leica, 39,601,006, USA). For fluorescence, liver specimens were embedded in OCT compound (Sakura, 4583, USA). Serum and liver samples were kept frozen at −70 °C until assayed.

### Animal groups and experimental design

For evaluation of the effect of Fuzheng Huayu Recipe (FZHY) against liver fibrosis, mice were randomly divided into 4 groups (12 mice per group): normal control, FZHY control, model control and FZHY treatment. From the third week of CCL4 injection, In the FZHY control and the FZHY treatment groups were orally administered Fuzheng Huayu Recipe at 5.6 g/kg daily, once a day for 4 weeks, which was equivalent to the dosage of a 60 kg adult. In the normal control and model control groups, mice were treated with ddH_2_O by gavage.

### Measurements of serum liver function and hydroxyproline content

The activities of serum alanine aminotransferase (ALT) and aspartate aminotransferase (AST) were quantitated by following the instructions provided by the manufacturer (Nanjing JianCheng Bioengineering Institute, Nanjing, China), including standardization. Levels of hydroxyproline (Hyp) in tissue were measured by Jamall’s method [16].

### Histopathological assessment of liver injury

Liver specimens were fixed in 10% formaldehyde solution and dehydrated in a graded alcohol series, embedded in paraffin blocks, and cut into 4 μm thick slices. For histopathological examination, slices were stained by using the standard procedure of haematoxylin and eosin (H&E). For investigation of the hepatic collagen deposition, Sirius red polarization staining was performed and red colour staining was considered to indicate collagen deposition. Images were analysed with a light microscope (Olympus BX40, Japan).

### Immunohistochemical analysis

Immunohistochemical analysis was performed to detect alpha smooth muscle actin (α-SMA) expression in liver tissues. In brief, paraffin-embedded sections were deparaffinized in xylene twice for 10 min, rehydrated in graded concentrations of ethanol (100%, 95%, 90%, 80% and 70%) and then were submerged in water. For antigen retrieval, the whole sections were submerged in citrate antigenic retrieval buffer and microwaved. They were then treated with 3% hydrogen peroxide in methanol to inactivate the endogenous enzymes. Sequentially, the sections were incubated with 5% bovine serum albumin (BSA) for 20 min at room temperature to block nonspecific binding. Sections were incubated with α-SMA (1:200, CST, D4K9N, USA) antibody overnight at 4 °C. After washing with TBST, tissue sections were treated with secondary anti-rabbit antibody (Boster, SA1028, China), followed by incubation with conjugated horse-radish peroxidase–streptavidin. Tissue sections were then counterstained with haematoxylin, dehydrated and mounted.Additional file: As per journal requirements, every additional file must have a corresponding caption. In this regard, please be informed that the caption of Additional file [1, 2] was taken from the additional e-file itself. Please advise if action taken appropriate and amend if necessary.Yes, the action taken is appropriate, thank you.

### Samples for RNA sequencing

RNA sequencing was conducted using homogenized liver tissue (three replicates each for the normal control, model control, and FZHY treatment). Total RNA was extracted using TRIzol reagent (Qiagen, Germany) according to the manufacturer’s instructions. RNA concentration, integrity, and quality were assessed using an Agilent Bioanalyzer system (Agilent, USA), with criteria for RIN values > 7 and 28 S:18 S ratio > 0.7. cDNA library construction using the Illumina TruSeqTM RNA Sample Prep Kit method. Subsequently, the Illumina NovaSeq 6,000 platform (LC Science, United States) was employed for quantification and sequencing according to a standard sequencing protocol. Quality control of raw sequencing data was performed using Trimmomatic software to generate clean data. Clean data were mapped to the mouse reference genome using HISAT2 (Rattus_norvegicus, version Rnor_6.0). Next, the gene FPKM expression value was quantified using cufflinks software. The read counts were normalized using DESeq2 software, the fold difference was calculated, and the significance of the difference was tested using the nbinomTest function, which used the default filter conditions (p-adjust < 0.05 and |log2 FC| ≥ 1).

### Quantification of retinoids by HPLC

Retinol metabolites were quantified in a blinded manner. All samples were frozen at collection and stored at −70 °C until extraction. Liver tissues were homogenized in saline and subjected to a 2-step liquid-liquid extraction under yellow lights as described previously. Levels of retinoic acid were determined by LC-MRM3 on a Shimadzu Prominence UFLC XR liquid chromatography system coupled to an AB Sciex 5500 QTRAP hybrid triple-quadrupole mass spectrometer using atmospheric pressure chemical ionization operated in positive ion mode. Retinol and retinal were quantified via HPLC-UV. Retinoic acid, retinol, and retinal were normalized per gram of tissue.

### Real-time fluorescence quantitative polymerase chain reaction

Total RNA was extracted using TRIzol Reagent (Sangon Biotech, Shanghai, China) in accordance with the manufacturer’s protocol. After the concentration of RNA was determined, 500 ng of total RNA was used as the template for reverse transcription into single-stranded cDNA by a Reverse Transcription Reagent Kit with gDNA Eraser (TaKaRa, Dalian, China). Real-time fluorescence quantitative polymerase chain reaction was performed using SYBR Premix Ex Taq (Tli RNaseH Plus; TaKaRa) and a ViiA 7 Real-Time PCR System (ABI, Carlsbad, CA, USA). The β-actin gene was amplified as an internal control, and the primer sequences are listed in Table 1. The relative gene quantities compared with β-actin were calculated through the 2^−∆∆CT^ method.Author contributions: Journal standard instruction requires the statement [All authors read and approved the final manuscript.] in the [Author contributions] section. This was inserted at the end of the paragraph of the said section. Please check if appropriate.We have verified it and resolved the issue, thank you.

**Table 1:** Primers for RT‒PCR

Gene	Forward (5′–3′)	Reverse (5′–3′)
**β-actin**	TGACGAGGCCCAGAGCAAGA	ATGGGCACAGTGTGGGTGAC
**Adh1**	TGTAAGCGTCGTCGTAGGAGTG	CTTAGCCATGAAGTCAGCCACAAG
**Adh3**	AGGAGTTGGACTGGCAGTGATC	TCTCAACGAGGACTTCCTGGATG
**Raldh1**	CATCACTGTGTCATCTGCTCTGC	CAGACATCTTGAATCCACCGAAGG
**Raldh2**	TGCGGATTGCCAAGGAGGAG	TTGCGGAGGATACCATGAGAGC
**Rae-1α**	ATGAAGCGAAGTGCTTAGTGGATG	GACACCTTGTTCCTCAACTTCTGG
**Rae-1γ**	CAAGGCAGCAGTGACCAAGC	CCACTAAGCACTTCGCTTCATACC
**Rae-1ε**	GCTGCAGTTCAAGACACCAA	TCCACTGAGCACTTCACGTC
**Rae-1δ**	CTGAGCTATGGATACACCAACG	AGCACTTCACTTCATCTGCTG
**Rar-α**	TTCTTTCCCCCTATGCTGGGT	GGGAGGGCTGGGTACTATCTC
**Rar-β**	AAGGGCTTTTTCCGCAGAAGT	GCATCGGTTCCTAGTGACCT
**Rar-γ**	ATGCACAAGGGTGACAACAG	GTCTCCACCGCTGAATGAAAA
**Rxr-α**	CTCAATGGCGTCCTCAAGGTTC	TGTTGTCTCGGCAGGTGTAGG
**Rxr-β**	CCACCTCTTACCCCTTCAGC	TGGAAGAACTGATGACTGGGA
**Rxr-γ**	CATGAGCCCTTCAGTAGCCTT	CGGAGAGCCAAGAGCATTGAG
**Cyp4a12a**	GTTTCAAGAGCCCTCCTAAGT	CAAAACAAGCATACACATTGG
**Cyp4a12b**	CAAGAGCCCTCCTAAGTGTTA	CAAAACAAGCATACACATTGG
**Alb**	GACGTGTGTTGCCGATGAGT	GTTTTCACGGAGGTTTGGAATG
**Desmin**	GTTTCAGACTTGACTCAGGCAG	TCTCGCAGGTGTAGGACTGG
**F4/80**	TGACTCACCTTGTGGTCCTAA	CTTCCCAGAATCCAGTCTTTCC
**Vwf**	CAATGGCACCGTAACGCAG	TGGAGAGCTTATAGTACCCAGC

### RNA in situ hybridization

RNA in situ hybridization was performed as follows. Briefly, liver samples were fixed in 4% paraformaldehyde in phosphate-buffered saline (PBS) at 4 °C overnight. Then the samples were embedded in Paraplast X-Tra (McCormic Scientific) and sectioned at a thickness of 8 μm using a microtome (Thermo Scientific). Sections were transferred to microslides and dried overnight at 42 °C on a slide warmer. Sections were dewaxed and incubated in pretreatment solution A for 30 min at room temperature to remove endogenous enzymes and then boiled in prereaction solution B containing citrate for 5 min. The mRNA nucleic acid to be tested was fully exposed by protease digestion at 40 °C for 10 min, and the gene-specific probe was incubated in the probe hybridization solution at 40 °C for two hours. After washing to remove unhybridized probes, signal amplification was performed using cascade signal amplification technology based on nucleic acid-protein hybridization (Chinese Patent No. ZL202110575231.5). Finally, fast red substrate was added for alkaline phosphatase-based colour reaction, and the position of target RNA is displayed as red dots or patch staining. The mRNA in situ detection of each gene used the PinpoRNATM RNA in situ hybridization detection kit (Guangdong Pinpoease Biotechnology product number PIT0001, https://www.pinpoease.com) manual operation detection. The probes used in PinpoRNA technology were designed with multiple sets of short probes for RNA detection (China Patent No. ZL202110581853.9) to ensure the specificity of the probes. The detection signal could only be displayed when it was combined with the mRNA to be detected at the same time. The designed probe covers the 283–747 base region of Cyp4a12a (Cat. No. IB1-00166).

### Immunofluorescence staining

The liver tissues embedded in OCT compound were cut into frozen sections at 10 μm in thickness. Then the sections were fixed in cold acetone for 30 min and treated with 0.1% Triton X-100 for 2 min at 4℃. The sections were blocked with normal donkey serum for 30 min at room temperature, before incubation with the α-SMA or retinoic acid early inducible gene 1 (Rae-1) (1:20, R&D, AF1136, USA). The tissues were then stained with Cy3 or FITC-conjugated secondary antibodies. After washing, DAPI (1:2000, Abcam, ab228549, UK) was used to stain the nuclei of the tissue. Images were taken with an Olympus confocal microscope, and semiquantitative analysis was performed using Image Pro Plus software.References: As per pubmed findings, citation details [Page no] for Reference [26] have been inserted. Kindly check and confirm the inserted details.We have conducted a thorough recheck of the reference and can confirm that it is in order. Thank you.

### Primary hepatocyte and HSC isolation, culture, and identification

Hepatocytes and HSCs were isolated from C57BL/6 mice by perfusing the liver through the inferior vena cava. The liver was perfused with EGTA buffer (136.89 mM NaCl, 5.37 mM KCl, 0.64 mM NaH_2_PO_4_.H_2_O, 0.85 mM Na_2_HPO_4_, 9.99 mM HEPES, 4.17 mM NaHCO_3_, 0.5 mM EGTA, and 5 mM glucose (pH 7.35–7.4)) at a rate of 5 mL/min for 2 min, followed by enzyme buffer (136.89 mM NaCl, 5.37 mM KCl, 0.64 mM NaH_2_PO4.H_2_O, 0.85 mM Na_2_HPO_4_, 9.99 mM HEPES, 4.17 mM NaHCO_3_, and 3.81 mM CaCl_2_.2H_2_O (pH 7.35–7.4)) containing 0.4 mg/mL pronase (Roche Diagnostics, Indianapolis, IN, USA) at a rate of 5 mL/min for 5 min, and then enzyme buffer containing 0.193 U/mg collagenase (Roche Diagnostics) at a rate of 5 mL/min for 7 min. After perfusion, the liver was shaken for 25 min at 37 °C and filtered through a 70 μm nylon mesh, and hepatocytes were pelleted by centrifugation at 500 rpm for 1 min at 4ºC. The supernatant was transferred and centrifuged at 580× *g* for 10 min at 4 °C. Pelleted HSCs were resuspended in Gey’s balanced salt solution (GBSS) (Sigma-Aldrich), gently overlaid with a gradient of Nycodenz (Alere Technologies, 1,002,424, USA) prepared with GBSS using a pipette, and then centrifuged at 1380 g for 17 min at 4 °C without braking. HSCs present in a thin white layer at the interface between Nycodenz and GBSS were harvested and washed with Hank’s balanced salt solution. The hepatocytes were plated in 1640 containing 20% foetal bovine serum (FBS), 10^− 8^ M dexamethasone, and 10^− 8^ insulin and HSCs were plated in DMEM containing 20% FBS.

### Oil red O staining

HSCs were stained with oil red O reagents (Nanjing Jiancheng, D027-1-2, China) according to the manufacturer’s instructions and the red lipid droplets were observed using a light microscope.

### Flow cytometry

After the mouse liver was removed, a mononuclear cell suspension was prepared by mechanical grinding. Percoll (Cytiva, 17,089,101, Sweden) was used for lymphocyte gradient separation. The cells were washed twice in PBS buffer containing 0.2% bovine serum albumin (BSA) and resuspended in PBS buffer for counting cell number with Trypan blue stain (Gibco, 15250-061, USA). Anti-mouse CD16/32 (eBioscience, 14-0161-86, United States) was used to block the Fc receptor. The following antibodies were used for immunophenotyping analysis of the samples: anti-mouse CD3-FITC (BD, 555,274, USA), anti-mouse NK1.1-APC (BD, 550,627, USA), and anti-mouse NKG2D-PE-Cy7(BD, 562,614, USA), Data were obtained by the flow cytometry (DxFLEX, Beckman Coulter, USA) and analysed using FlowJo 10.0 software.

### Primary NK cell isolation, culture, and identification

Murine primary NK cells were isolated from Percoll-separated hepatic mononuclear cells by magnetic sorting using an NK cell isolation kit (Miltenyi,130-115-118, Germany). NK cells were sorted as cells that expressed NK1.1^+^CD3^−^ using flow cytometry, and the purity was higher than > 95%. After purification, NK cells were cultured in a 96 round bottom wells in complete RPMI 1640 medium supplemented with 10% (v/v) foetal bovine serum (Hakata, HB-FBS-500, Japan), 100 units/ml penicillin(Sangon Biotech, A100339, China), 100 µg/ml streptomycin (Sangon Biotech, A100382, China) and interleukin-2 (IL-2, 10 U/ml, R&D,402-ML, USA).

### Drug incubation

FZHY was initially dissolved as a concentrated stock solution in DMSO. For evaluation of the effect of FZHY on HSCs in vitro, HSCs were treated with 8 concentrations (6.25, 12.5, 25, 50, 100, 200, 400,800 µg/ml) of FZHY, for a period of 24 h.

### Coculture of NK cells and HSCs

HSCs were cultured in adherent 96 flat bottom wells for 24 h. and the empty medium containing the FZHY drug was disturbed. After the drug-containing supernatant was discarded, primary NK cells were added to a 96-well plate (at a ratio of 10:1).

### Cell viability assay

For the HSCs, cell viability assays were performed by using Cell Counting Kit-8 (CCK8, MCE, HY-K0301, USA) assays following the manufacturer’s suggestions.

### Calculation of percent lysis

The CellTiter-Glo^®^ Luminescent Cell Viability Assay kit (Promega, G7570, USA) was used to calculate percent cocultured cell viability using the mean luminescence signal of a mixture of NK cells and HSCs (MeanMIX) minus the mean luminescence signal of NK cells (MeanNK) divided by the mean signal of HSCs (MeanHSC) according to the methods of a previous study [17].

### Adenovirus transfection to silence genes

The recombinant adenovirus shuttle vector Hu6-MSC-CMV-EGFP for silencing CYP4a12a short hairpin RNA (shRNA) (AD-Cyp4a12a-RNAi) was constructed by GeneChem Co. Ltd (Shanghai, China). The optimal sequence for knockdown of CYP4a12a (5′-GGAACATCTTTCACCAGAATG-3′) was selected and the scrambled sequence (TTCTCCGAACGTGTCACGT) was used as a negative control. After 48 h of infection, HSCs with green fluorescence were recorded. The knockdown efficiency of shRNA was determined by real-time PCR.

### Statistical analysis

All data were analysed by using PASW Statistics 18 software. Differences between the groups were assessed by nonparametric one-way analysis of variance (ANOVA) followed by the least significant difference (LSD) post hoc tests. Values in the text are means ± standard deviations (SD). Differences with *p* < 0.05 were considered to be statistically significant.

## Results

### FZHY effectively alleviated CCL _4_ -induced liverfibrosis in mice

**Fig. 1:**
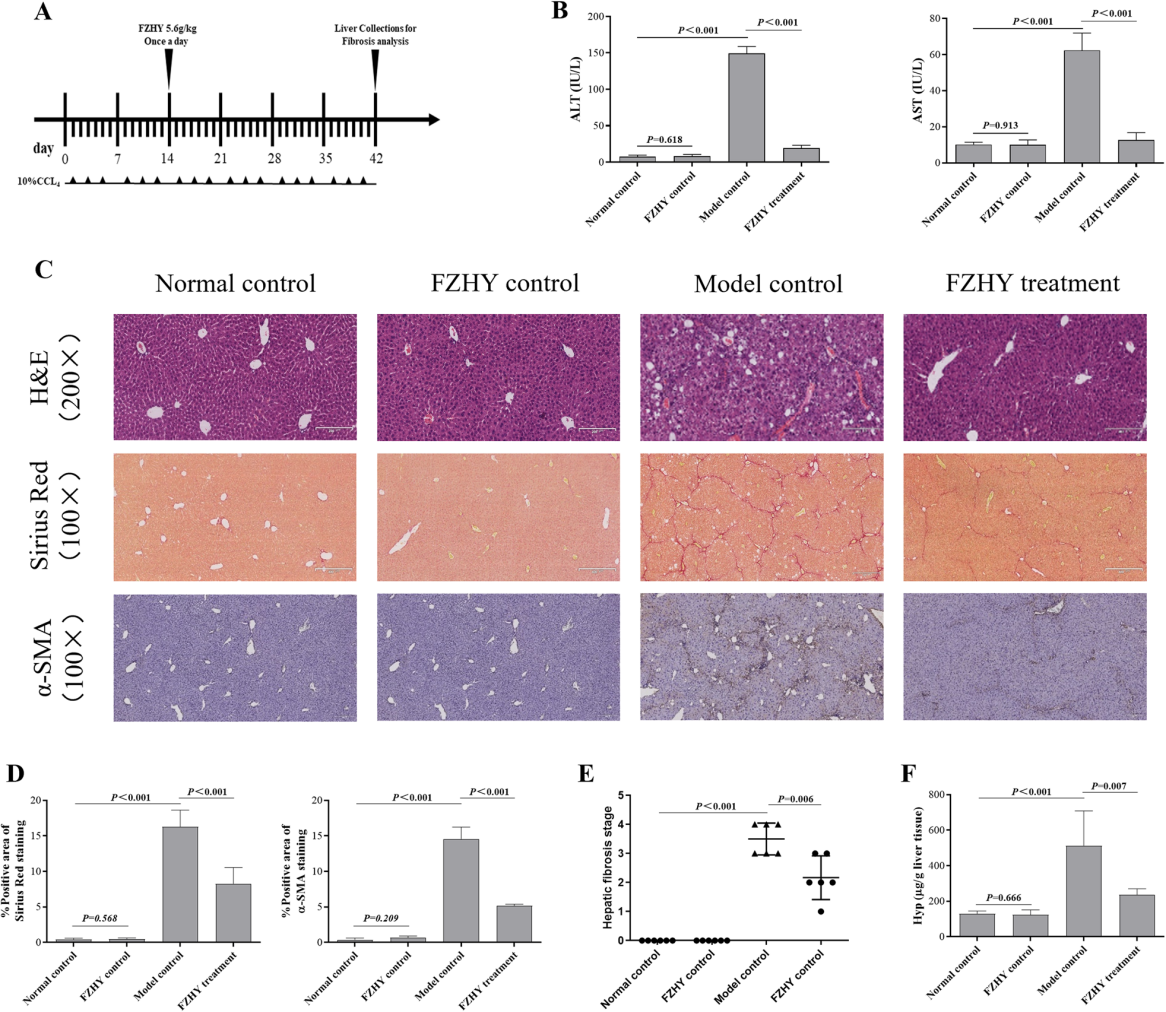
FZHY effectively reverses CCL4-induced liver fibrosis in mice. **A** Six-week-old mice were injected intraperitoneally with 10% CCL4 at a dose of 2 ml/kg, three times a week for 6 weeks. After 2 weeks of CCL4 injection, daily treatment with FZHY at 5.6 g/kg began. The normal control group received vehicle as control. Six weeks later, the mice were sacrificed 48 h after the last CCL4 injection. **B** Serum levels of serum ALT and AST were assayed by using commercial kits. **C** Representative images of H&E staining, Sirius red staining and immunohistochemical staining (H&E staining, magnification 200x, Sirius red and immunohistochemical staining, magnification 100x). **D** Semiquantification data were obtained by analysing Sirius red staining and immunohistochemical staining liver sections. Data were acquired using Image-Pro Plus software. **E** Liver fibrosis staging data assessed by Sirius red staining which using Redit analysis. **F** Hyp contents were quantified from 100 mg liver samples and were measured by Jamall’s method. Values represent the means ± SDs

A model of CCL_4_-induced liver fibrosis was developed, and the effects of FZHY on liver fibrosis were investigated. CCL_4_ was administered intraperitoneally to mice for 6 weeks, and FZHY was administered orally beginning in the third week (Fig. 1A). Serum transaminase levels in the model control group were higher than those in the normal control group, but they were significantly improved after FZHY treatment (Fig. 1B). Figure 1C–E displays representative images of H&E, Sirius red, and α-SMA staining of the four groups. After FZHY treatment, the inflammatory infiltrations were improved, and the fibrous septa and α-SMA expression were significantly reduced (Fig. 1C–E). The Hyp content was higher in the model control group than those in the normal control group, but significantly improved in the FZHY treatment group (Fig. 1F). Overall, FZHY could alleviated CCL_4_- induced liver fibrosis.

### Transcriptomic data of FZHY intervention in CCL _4_ -induced liver fibrosis in mice

**Fig. 2:**
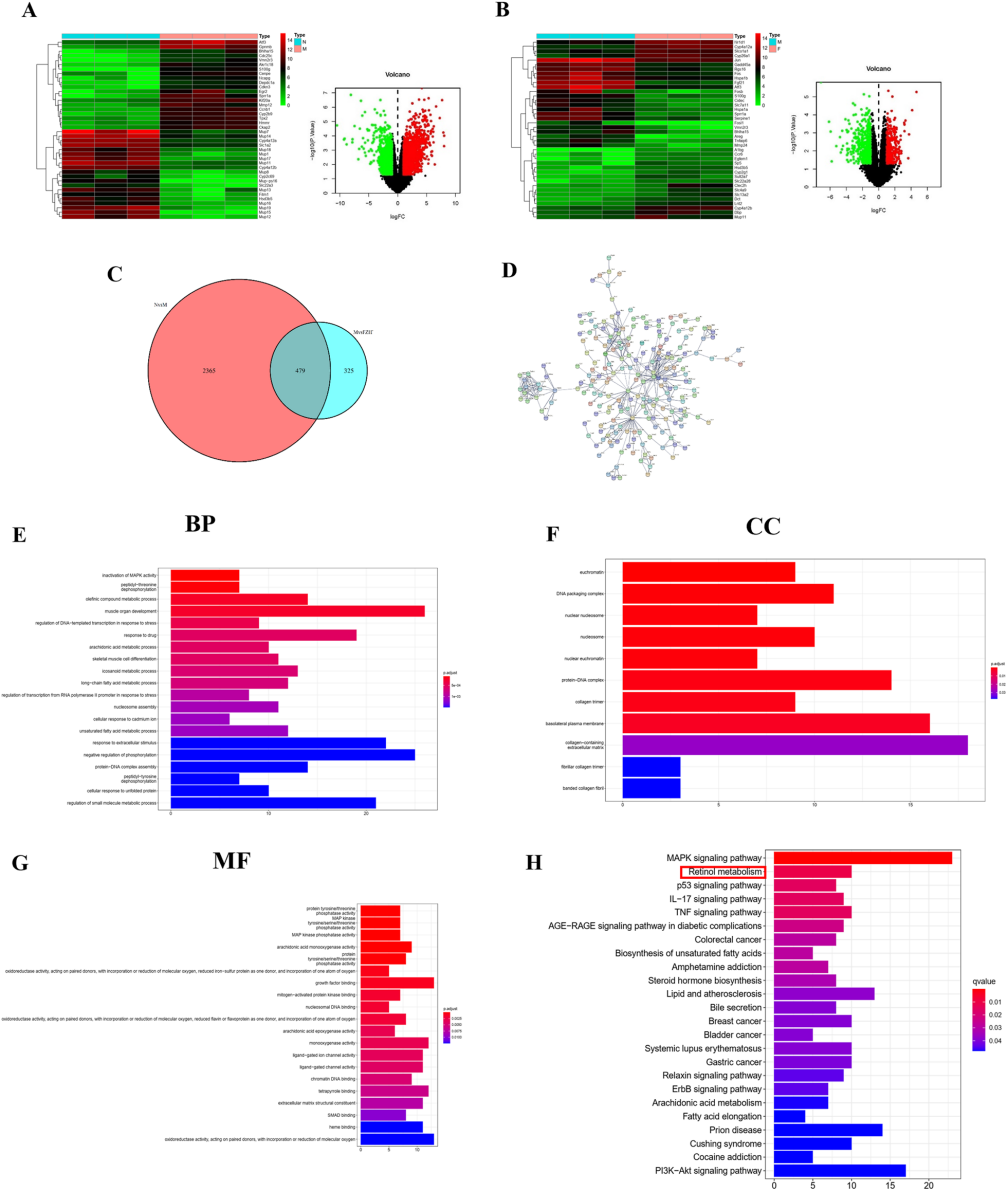
Transcriptomic data of FZHY intervention in CCL4-induced liver fibrosis in mice. **A** Heatmap and volcano plot of different genes in normal control vs. model control. **B** Heatmap and volcano plot of different genes in model control vs. FZHY treatment. **C**–**D** Venn maps and PPI network diagram of differentially expressed genes in normal control vs. model control vs. FZHY treatment. In addition, the top 30 differentially expressed genes are exhibited. **E**–**G** The GO analysis of cross-target genes of FZHY intervention in CCL4-induced liver fibrosis in mice. **H** KEGG pathway enrichment analysis of cross-target genes; the pathway enrichment results include retinol metabolism (marked with a red square line)

To confirm the mechanism of action of the FZHY recipe, we used animal liver tissue samples for transcriptomic analysis to generate RNA-seq data from three biological groups: normal control, model control, and FZHY treatment. The differential gene changes in the model group compared with the normal control group were obvious. A total of 1597 genes were upregulated and 1247 genes were downregulated in the model group (Fig. 2A, Additional file 2: Table S1). Furthermore, 324 genes were upregulated and 480 genes were downregulated in the treatment groups compared with the model control group (Fig. 2B, Additional file 2: Table S2). The Venn diagram shows a total of 479 unique and coexpressed genes between the two differential groups (Fig. 2C, Additional file 2: Table S3). Interactions between differentially expressed genes were visualized by a protein-protein interaction network (Fig. 2D). To obtain an overview of the effects, we performed Gene Ontology (GO) analysis for functional categorization of the differentially expressed genes. Figure 2E–G and Additional file 2: Tables S4–S6 indicate the molecular function category (MF), cellular component category (CC) and biological process category (BP) of the differentially expressed genes. Next, we performed pathway analysis using the KEGG pathway database. Interestingly, we found that the retinol metabolism pathway was enriched (Fig. 2H, Additional file 2: Table S7).

### The Cyp4a12 was involved in the regulation of retinoic metabolism interfered wity by FZHY in vivo

**Fig. 3:**
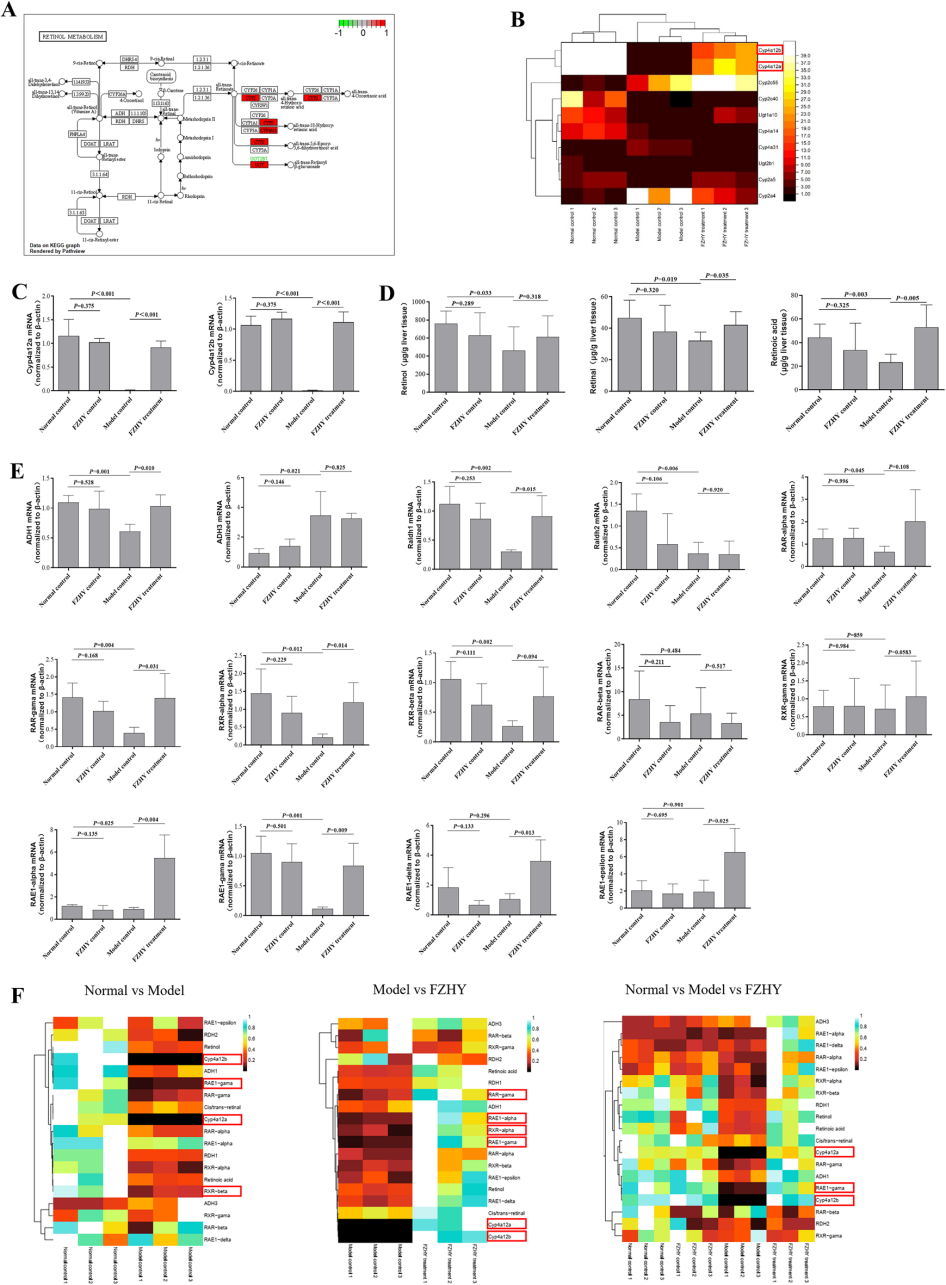
Cyp4a12 was involved in the regulation of retinoic metabolism interfered by FZHY in vivo. **A** The enriched genes in the retinol metabolism. **B** A heatmap showing the relative intensity of retinol metabolism-related markers in the KEGG analysis. **C** The mRNA levels of CYP4A12a/b in liver. **D** Mass spectrometry of retinol, retinal and retinoic acid in liver. **E** The relative mRNA expression levels of retinol metabolism-related genes in liver. **F** A heatmap showing the relative intensity of retinol metabolism-related markers in the clustering analysis, in the normal control vs. model control vs. FZHY treatment. Values represent the means ± SDs

The liver is an important site for the storage of retinol substances in the body. To investigate the specific effect of FZHY on the retinol metabolism, we generated a KEGG graph of the retinol metabolism signalling pathway was presented. As previously described, the roles of CYP26 and CYP2 in RA metabolism were shown. Notably, the regulation of Cyp4a11 (designated as Cyp4a12a/b in mice) was highlighted (Fig. 3A–B). Therefore, we first examined the changes in Cyp4a12 in the liver in each group. Currently, two Cyp4a12 genes, Cyp4a12a and Cyp4a12b, have been identified [18] The mRNA levels of Cyp4a12a and Cyp4a12b (CYP4A12a/b) were decreased in the model control group and increased in the FZHY treatment group (Fig. 3C). Since retinol is hydrolysed to retinal and then metabolized to retinoic acid under the action of alcohol dehydrogenase (Adh) and retinal dehydrogenase (Raldh), we detected the concentrations of retinol, retinal, and retinoic acid in liver tissues by mass spectrometry. In addition, the mRNA levels of Adh and Raldh were assayed by RT-PCR. As shown in Fig. 3C and D, the levels of hepatic retinol, retinal and retinoic acid were decreased in fibrosis mice, whereas the content of Adh3 was increased. After FZHY treatment, the expression levels of Adh1, Raldh1 and Raldh2 were significantly increased with the reduced Adh3 (Fig. 3C and D). Next, we detected Rars and Rxrs, respectively, both of which have 3 isoforms known as alpha, beta and gamma [19]. Rarα, Rarβ, Rxrα, and Rxrγ mRNA levels were shown to be decreased in the model control group, and FZHY restored only the Rar and Rxr levels (Fig. 3E). Additionally, it was noted that Rae-1α and Rae-1γ expression was downregulated in the model control group, and FZHY was able to restore Rae-1α and Rae-1γ expression (Fig. 3E). All of the genes impacted by the retinol metabolic pathway were analysed using heatmaps, with Cyp4a12a/b and the metabolite Rae-1 being the most noticeable modifications (Fig. 3F). In conclusion, the Cyp4a12 gene is involved in retinoic acid metabolism and affects Rae-1, both of which are regulated by FZHY.

## The retinol metabolism of HSCs was influenced by FZHY in vivo

**Fig. 4:**
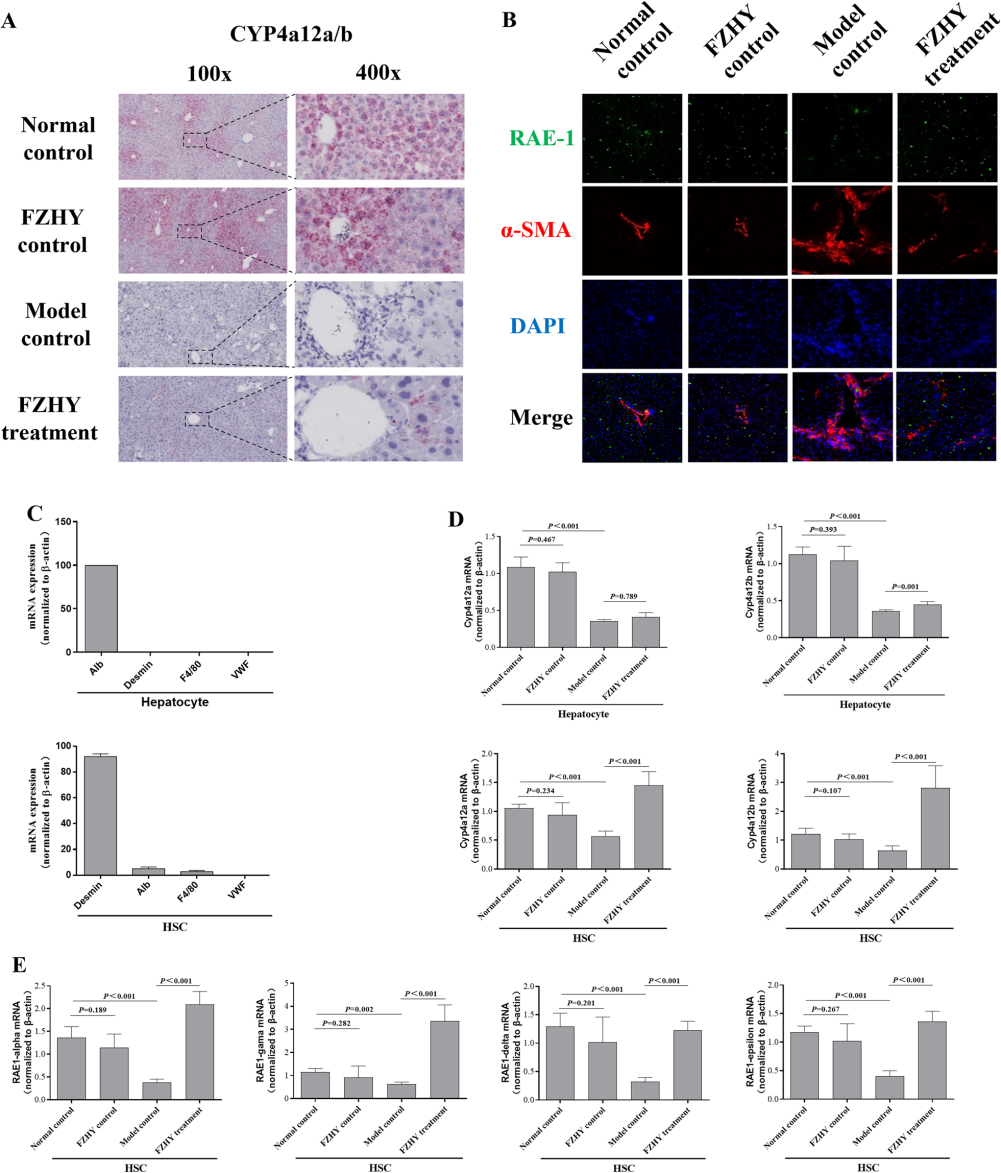
The influence of retinol metabolism pathway mainly comes from HSCs in vivo. **A** The Cyp4a12a/b enzyme of liver tissue were stained with Pinpo RNA in situ hybridization. **B** The immunofluorescence staining of α-SMA (red immunofluorescence) and RAE-1 (green immunofluorescence). **C** Primary HSCs and hepatocytes were isolated from mice with CCL4-induced liver fibrosis were identified by RT‒PCR for Desmin, Alb, F4/80, Vwf. **D** The relative mRNA expression levels for Cyp4a12a/b were determined from HSCs and hepatocytes. **E** The relative mRNA expression levels for RAE-1 were determined from HSCs. Values represent the means ± SDs

In situ hybridization to observe Cyp4a12a/b and immunofluorescence labelling to detect Rae-1 were utilized to determine the cellular source of Cyp4a12 in the liver. Significant decreases in Cyp4A12 and Rae-1 were detected in model controls, as predicted, and this effect was altered by FZHY (Fig. 4A and B). Unfortunately, although Cyp4A12 alterations in liver fibrosis have been observed, we cannot confirm the origin of the cells because they were extensively distributed in hepatic sinusoids and central veins. As a result, the parallel approach was repeated in identify primary HSCs (ester storing cells) and hepatocytes (highly expressing cytochrome P450). The purity of the cells was validated by utilizing Desmin (a marker for HSCs), F4/80 (a marker for macrophages), VWF (a marker for endothelial cells), and Alb (a marker for hepatocytes) (Fig. 4C). Cyp4a12a/b levels were shown to be lower in HSCs and hepatocytes, respectively. However, FZHY was only exclusively effective in HSCs and had no impact on hepatocytes (Fig. 4D). We concluded that the facilitation effect of FZHY on Cyp4a12 metabolism occurred primarily in HSCs. Furthermore, FZHY treatment dramatically increased the mRNA levels of Rae-1 (α, β, δ, ε) in HSCs (Fig. 4E). Therefore, we hypothesized that FZHY might affect retinol metabolism by influencing Cyp4a12 expression in HSCs.

## Retinol metabolism in HSCs was regulated by FZHY treatment in vitro

**Fig. 5:**
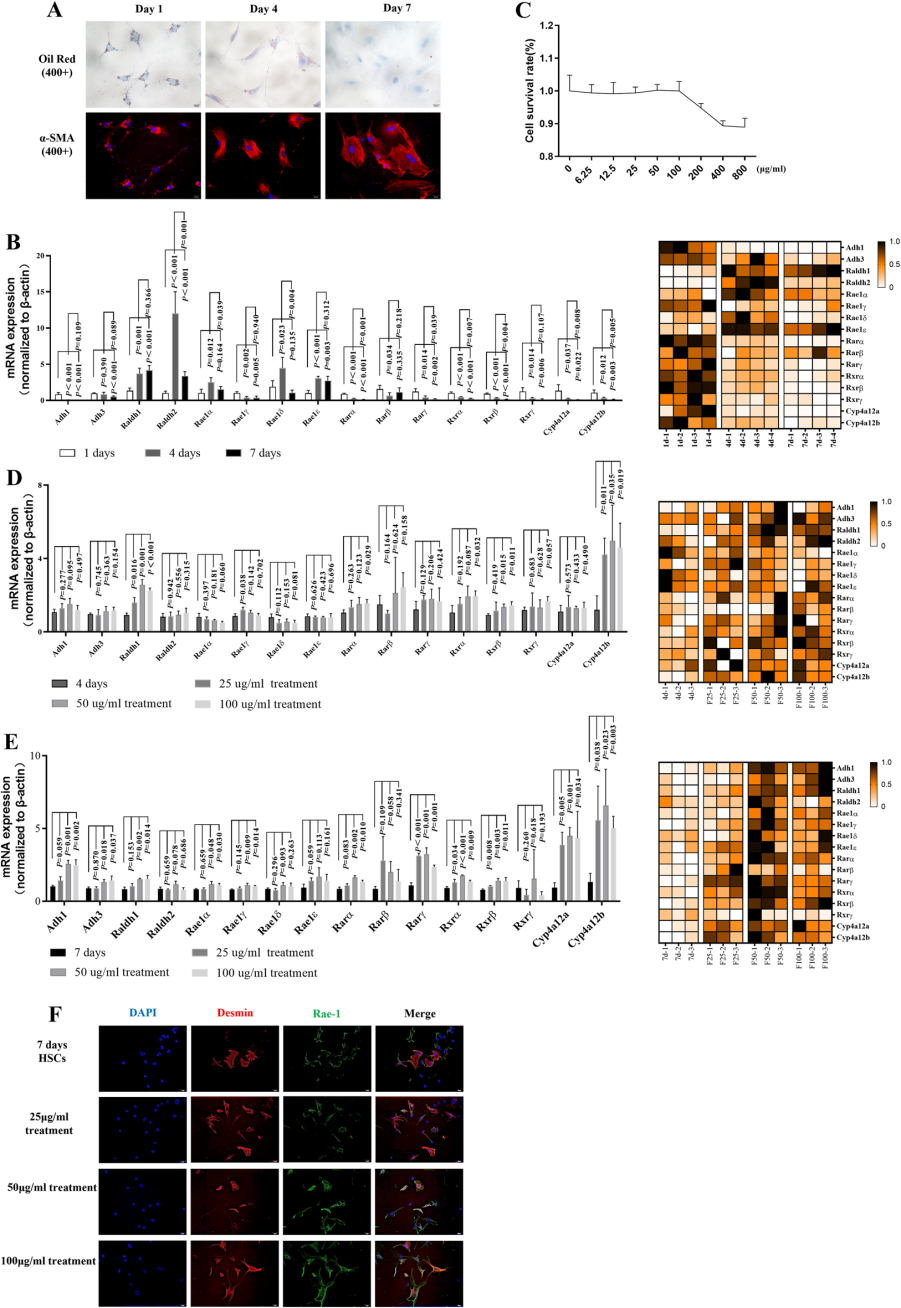
The retinol metabolism in HSCs was regulated by FZHY treatment in vitro. Primary hepatic stellate cells were isolated and cultured in vitro through normal C57BL/6 mice, which were spontaneously activated on Day 1, Day 4, and Day 7. **A** Oil red staining and immunofluorescence staining of α-SMA (red immunofluorescence) in HSCs. **B** Dynamic observation of the relative mRNA expression levels of retinoic acid metabolism-related genes in HSCs. **C** Determination of a nontoxic concentration of FZHY. **D**–**E** Pharmacodynamic observation of the relative mRNA expression levels of retinoic acid metabolism-related genes in HSC cultured for 4 days and 7 days. **F** The immunofluorescence staining of Desmin (red immunofluorescence) and Rae-1 (green immunofluorescence). Values represent the means ± SDs

We further explored the dramatic changes in Cyp4a12a/b during HSC activation. Primary HSCs were isolated from normal murine livers and cultured for 1 (quiescent period), 4 (intermediate period), and 7 (active period) days, respectively [20], in order to mimic the similar process of HSC activation in vivo. Because of the progressive activation of HSCs, the lipid droplet content decreased in a day-dependent manner (Fig. 5A). We found that retinoic acid metabolism in HSCs was altered dynamically, with the gradual declines in Adh1, Rar-α/γ, Rxr-α/β/γ and Cyp4a12a/b levels. Raldh1/2 mRNA levels, on the other hand, were highly expressed in HSCs on Day 4. Furthermore, Rae-1 mRNA expression was upregulated in 4-day cultured HSCs and considerably decreased in 7-day cultured HSCs (Fig. 5B), consistent with previous reports [15]. Then, the effect of FZHY on retinol metabolism in HSCs was observed. FZHY were processed for HSCs cultured for 4 days and 7 days. Figure 5C shows the maximum nontoxic concentration of FZHY to be approximately 100 µg/ml. Further experiments were performed using concentrations lower than the maximum nontoxic concentrations (25, 50, 100 µg/ml). Interestingly, real-time PCR analysis showed that the changes of retinoic acid metabolism were not appreciable in FZHY-treated HSCs at 4 days compared with 7-day HSCs, which suggested that FZHY could restore the expression of Cyp4a12 and Rae-1 in fully activated HSCs (Fig. 5D–E). Immunofluorescence staining showed the same results (Fig. 5F). Overall, our results suggest that the retinol metabolism in HSCs was regulated by FZHY treatment in vitro.

## Cyp4a12/b is the key regulator of FZHY treatment via regulation of Rae-1

**Fig. 6:**
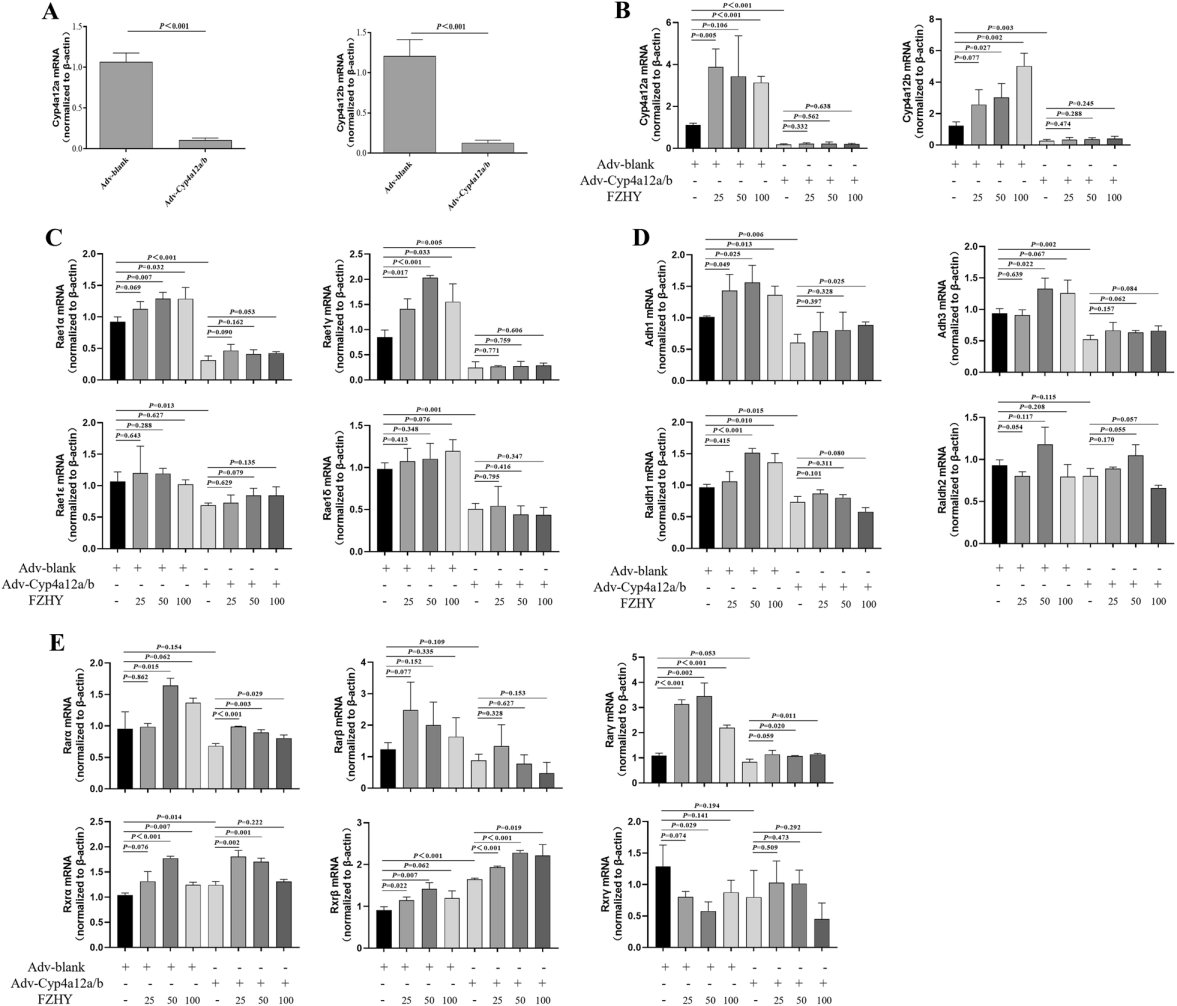
The Rae-1 expression was restored under the FZHY treatment by regulating Cyp4a12a/b in vitro. HSCs were isolated from healthy control mice and adapted in culture for 1 day, and then, adenovirus was used to transfect 1 day HSCs for 48 h, as described in Materials and Methods. **A** The Cyp4a12a/b inhibition rates were observated through quantitative real-time PCR assays. **B** Pharmacodynamic observation of Cyp4a12a/b mRNA expression levels in adenovirus-transfected HSCs. **C** Pharmacodynamic observation of Rae-1α/γ/δ/ε mRNA expression levels in adenovirus-transfected HSCs. **D**, **E** Pharmacodynamic observation of relative mRNA expression levels of retinoic acid metabolism-related genes and nuclear receptors in adenovirus-transfected HSCs. Values represent the means ± SDs

From the results above, Cyp4a12a/b was a potential target for regulating retinol metabolism in HSCs, and whether FZHY exerts a pharmacological effect by targeting Cyp4a12a/b was investigated. Then, Cyp4a12a/b expression was silenced by adenovirus-mediated gene modification. Adenovirus was used to transfect 1-day HSCs for 48 h (Additional file 1: Figure S2A), and a 90% inhibition rate of the Cyp4a12a/b gene was observed (Fig. 6A). Interestingly, Oil red O staining showed that silencing Cyp4a12a/b expression did not alter the basal levels of lipid droplets (Additional file 1: Figure S2B); however, the mRNA levels of retinoic acid metabolic enzymes and nuclear receptors were affected (Fig. 6D–E), and the expression of Rae-1 was significantly suppressed (Fig. 6C). Then, HSCs infected with adenovirus were treated with FZHY. Cyp4a12a/b and Rae-1 were upregulated in HSCs treated with FZHY compared to those treated with adenovirus-blank (Fig. 6B–C). These results indicated that Cyp4a12a/b was a crucial regulatory pathway for Rae-1 by FZHY. However, adenovirus transfection prevented the regulatory effect of FZHY treatment on increases in Cyp4a12a/b and Rae-1 (Fig. 6B–C). This phenomenon did not appear to affect the mRNA levels of retinoic acid metabolic enzymes or nuclear receptors. In conclusion, these results suggest that FZHY contributes to the restoration of Rae-1 expression in vitro by regulating Cyp4a12a/b.

### FZHY promotes NK cell regains recognition and killing of primary HSCs

**Fig. 7:**
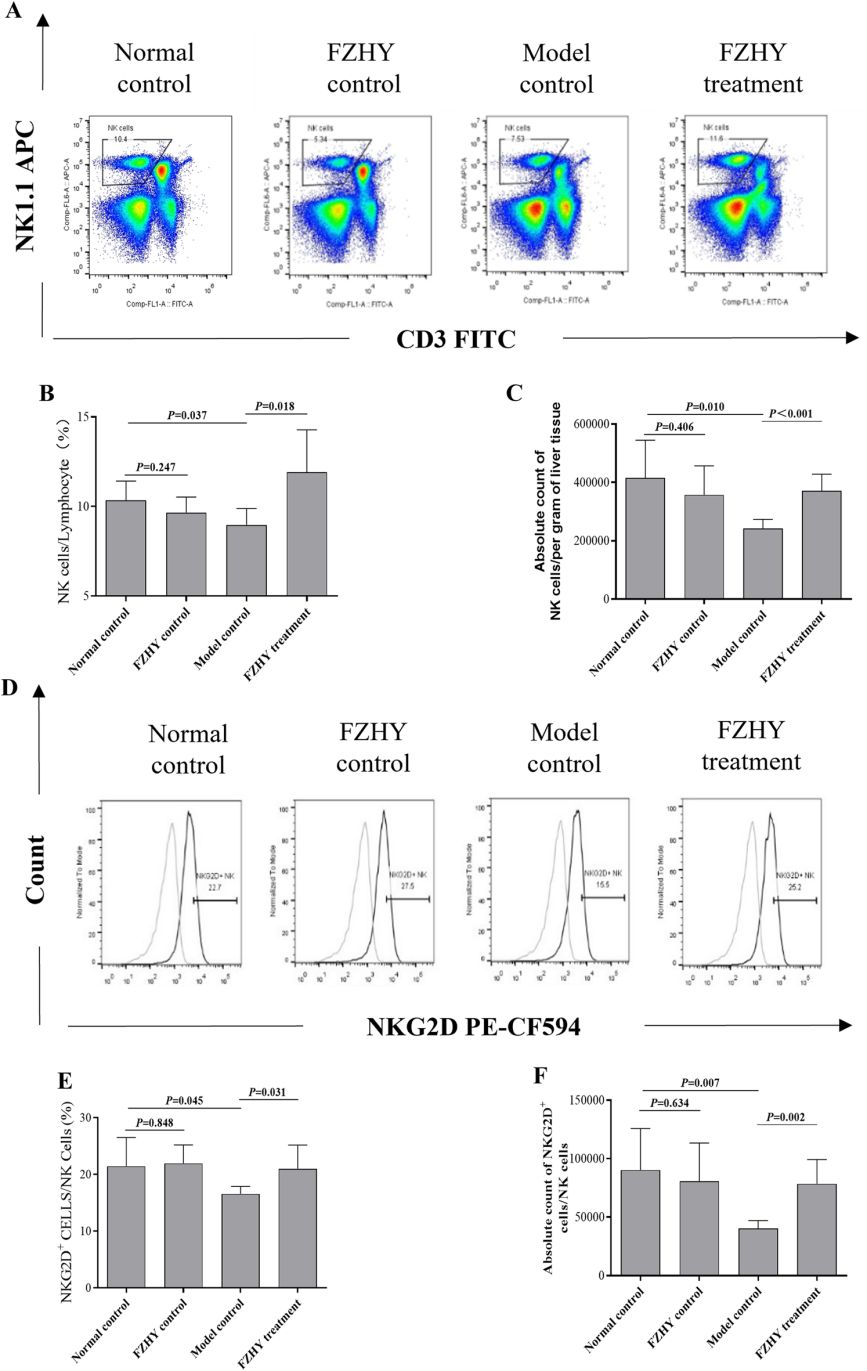
FZHY promotes NKG2D upregulation of liver NK cells in vivo. Lymphocytes were isolated from the livers of CCL4-injected mice, and analysed by FACS with APC-conjugated rat anti-mouse NK1.1 antibody, PE-Cy7-conjugated rat anti-mouse CD3 monoclonal antibody and PE-CF594-conjugated rat anti-mouse NKG2D monoclonal antibody. **A**–**C** Analysis of liver NK-cell proportions and count. **D**–**F** Frequency and number of NKG2D^+^cells in liver NK-cell were counted. Valu Values represent the means ± SDs

The Rae-1 genes have been identified as ligands for mouse NKG2D, an NK cell-activating receptor known to be expressed on NK cells and certain subsets of T cells [21]. We therefore determined NKG2D expression on NK and CD3^+^ T cells. Additional file 1: Figure S3A shows the representative gating strategy used to analyse lymphocyte populations within livers (Additional file 1: Figure S3A). Decreases in NK cells and CD3^+^ T cells were observed in the model control, whether in terms of relative frequency or absolute counts (Fig. 7A, Additional file 1: Figure S3B). Then, we observed that NKG2D expression was decreased on NK cells and increased on T cells (Fig. 7B, Additional file 1: Figure S3C). Treatment with FZHY reversed all of these effects on NK cells and had no appreciable effect on CD3^+^ T cells.

**Fig. 8:**
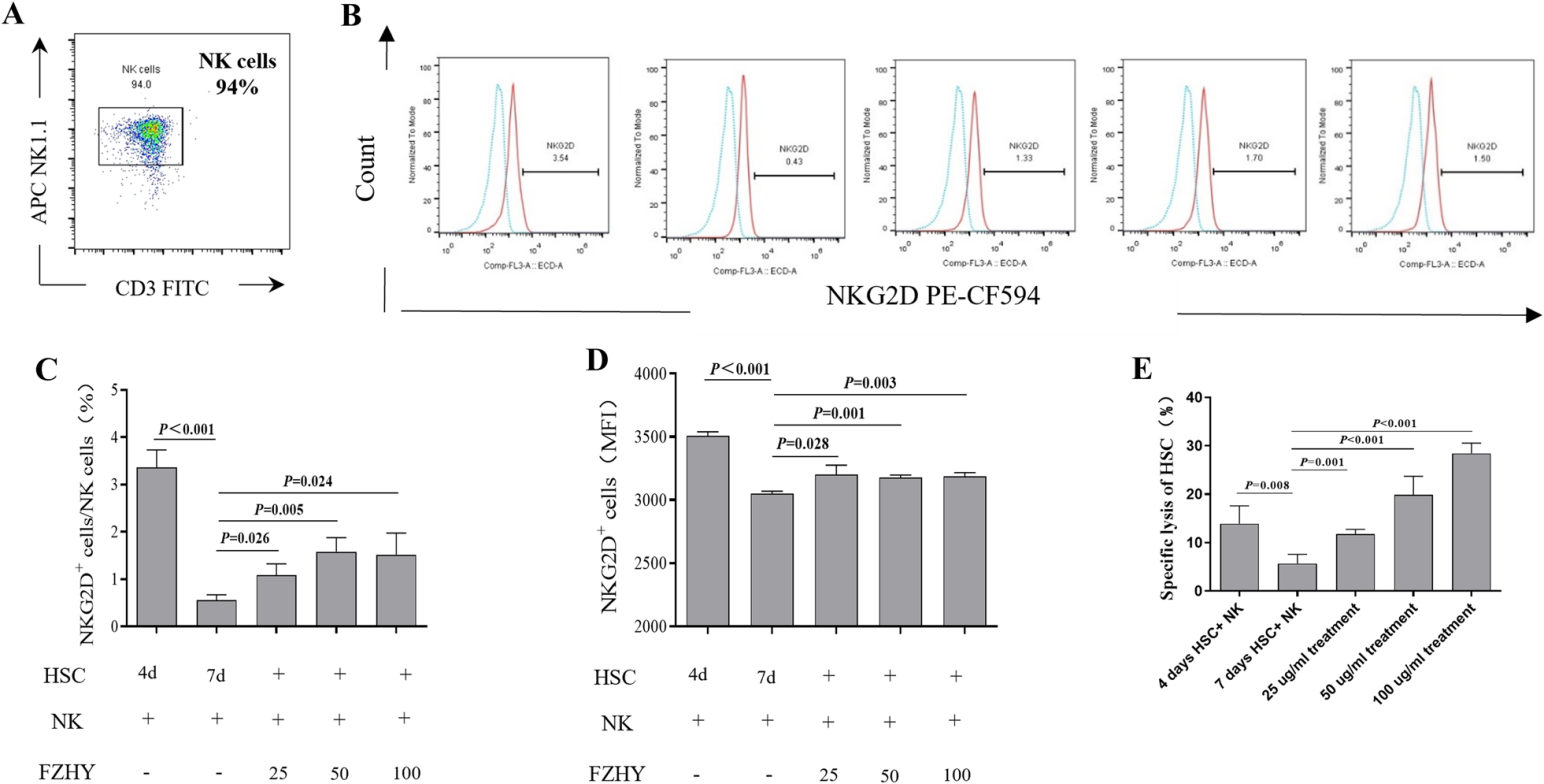
FZHY promotes recognition and killing of primary HSCs of NK cells. HSCs cultured for 4 days and 7 days in vitro were cocultured with primary NK cells at a 1:10 effector-target ratio. **A** Primary NK cells were isolated in vitro from normal C57BL/6 mice and were identified by flow cytometry. **B**–**D** The frequency and MFI of NKG2D^+^ cells were determined. **E** The specific lysis rate of HSC in the coculture system was determined. Valu Values represent the means ± SDs

To further confirm the effect of Rae-1 variation on the ability of NK cells to kill HSCs, we used in vitro coculture experiments. NK cells were isolated from the livers of normal mice. By flow cytometry, CD3^−^NK1.1^+^ cells had a purity > 90% (Fig. 8A). NK cells were seeded into plates where HSCs were preincubated in the absence or presence of FZHY for 24 h (target ratios 10:1). Evaluation of NKG2D expression by flow cytometry showed that activated (7-day) HSCs induced NK cells to produce less NKG2D, and NK cell cytotoxicity to HSCs was decreased compared with that of the 4-day HSCs (Fig. 8B–E). FZHY treatment promoted NKG2D expression in a dose-dependent manner. Moreover, the cytolysis of HSCs was increased. The reduction in surface expression of Rae-1 in activated HSCs (Fig. 5) helped the cells evade recognition and killing by NK cells. FZHY could restore NK cell cytotoxicity via Rae-1/NKG2D binding by modulating retinoic acid metabolism. Overall, FZHY promotes NK cell recognition and killing of primary HSCs by increasing Rae-1 expression.

## Discussion

Liver fibrosis is a dynamic process in chronic injury characterized by the persistent accumulation of fibrillar ECM associated with simultaneous matrix degradation and remodelling [22]. Activated HSCs are considered to be key effectors that increase the deposition of fibrillar ECM. Among the numerous biochemical changes in the activation of HSCs, the loss of lipid droplets is the most striking phenomenon. The vast majority of retinoids in humans are stored in lipid droplets in the cytoplasm of HSCs as retinyl esters. Activated HSCs are accompanied by loss of intracytoplasmic lipid droplets as well as activation of retinoic acid signalling. Retinol is oxidized to retinaldehyde, catalysed by alcohol dehydrogenase, and then oxidized to RA, which is catalysed by Raldh. RA signalling is mediated by two subfamilies of nuclear retinoid receptors: the RA receptor (RAR) subfamily, which comprises RARα, RARβ and RARγ, and the RXR subfamily, which comprises RXRα, RXRβ and RXRγ, which regulates target gene transcription by binding to retinoic acid-responsive elements in DNA. Previous studies have suggested that the activation of retinoic acid signalling is associated with fibrotic development. Retinol deficiency is considered an independent risk factor associated with liver fibrosis in the reversible stage of liver disease (NAFLD) [23]. RA can inhibit liver fibrosis by inhibiting type I collagen production and reducing oxidative stress-induced liver damage [24]. Shimizu H et al. [25] also found that RA reduced the mRNA levels of α-SMA and type I collagen in HSCs. Furthermore, Rae-1 produced by retinoic acid metabolism was confirmed to be expressed on the surface of activated HSCs at an early stage, triggering increased NKG2D expression [7]. Accordingly, further examining retinoic acid mechanisms in liver fibrosis is likely to have a significant impact.

Cytochrome P450 (CYP) is a membrane-bound, heme-containing oxidoreductase that is a part of the multienzyme complex and has been confirmed to participate in multiple HSC metabolic processes, including retinol metabolism, central carbon metabolism and redox [26]. All studies, however, predominantly focused on CYP26 and CYP2C, and relevant studies on CYP4As are seldom reported. Here, we found several members of the CYP4A subfamily involved in retinol metabolism via transcriptome sequencing and investigated the mechanisms underlying this process. The results of the in vivo experiment showed that the expression of Cyp4a12a/b was dramatically reduced during the development of fibrosis, while retinoic acid metabolic pathways were regulated, resulting in decreases in Rae-1. These results indicated that the effect of Rae-1 might be attributed to Cyp4a12a/b-regulated retinol metabolism. Indeed, the in vivo results of the present study were further confirmed in vitro. The content of Cyp4a12a/b was gradually lost during HSC activation, and the expression of Rae-1 was reduced. Adenoviral knockdown of Cyp4a12a/b in HSCs also led to decreased Rae-1 expression. Taken together, the results suggest that changes in Rae-1 were ascribed to the regulation of Cyp4a12a/b. We further investigated the association between the effect and liver fibrosis. Given that Rae-1 was identified as a mouse NKG2D ligand [27], we next examined NKG2D-expressing NK cells and subsets of T cells among the lymphocytes of the liver. We found that NKG2D expression decreased in NK cells, while increased or decreased Rae-1 in HSCs could directly influence the cytotoxicity of NK cells. From this, we suggest that the drastic reduction in Cyp4a12a/b impacts retinol metabolism, resulting in a reduction in the production of Rae-1 and, in turn, the capability of clearance by NK cells to activate HSCs. Notably, this effect was associated with antihepatic fibrosis.

Fuzheng Huayu recipe (FZHY) is a traditional herbal medicine that has been administered to patients with hepatic fibrosis and cirrhosis. FZHY has been shown to exert antifibrotic effects by targeting multiple molecules of the immune microenvironment of the liver [28]. Zhang M et al. [29] found that FZHY could prevent and treat liver fibrosis by regulating the polarization and chemotaxis of intrahepatic macrophages via CCL2 and CX3CL1. Wu M et al. [13] reported that FZHY inhibited the activation of HSCs to attenuate hepatic fibrosis. In our research, based on the regulation of Cyp4a12a/b, we explored the effects of FZHY on retinol metabolism in HSCs. Overall, our results corroborate that Cyp4a12a/b from HSCs rather than from hepatocytes with high cytochrome P450 expression is mainly regulated by FZHY. Rae-1 was restored to enhance NK cell cytotoxicity by regulating retinol metabolism.

In conclusion, Cyp4a12a/b-mediated retinol metabolism in HSCs is the antihepatic fibrosis mechanism of FZHY. However, the metabolic processes of Cyp4a12a/b in the body are unclear, and elucidation of the mechanistic basis of Cyp4a12a/b will offer further insight into developing drugs for the prevention and treatment of liver fibrosis.

## Supplementary Information

**Additional file 1: Figure S1. **The chromatographic profile of FZHY extracts, flow rate: 1 mL/min). Peak No.: 1. danshensu; 2. protocatechuic aldehyde; 3. rosmarinic acid; 4. salvianolic acid B; 5. schizandrol A; 6. schizandrol B; 7. schizandrin A; 8. uridine 9. guanosine; 10. adenosine. **Figure S2. **HSCs were transfected with and without adenoviral vector for Cyp4a12a/b.Adenovirus was used to transfect 1 day HSCs for 48h and transduc-tion at MOI 1000 was the best results. Oil red staining in adenovirus-transfected HSCs was observed. Values represent means ± SD. **Figure S3. **The effects of FZHY on CD3+T cells expressed NKG2D.Gating strategy for flow cytometry analysis.Analysis of liver CD3+T-cells proportions and count.Frequency and number of NKG2D+-cells in liver CD3+T-cells were counted. Values represent means ± SD.

**Additional file 2: Table S1. **The differential gene changes in Normal control vs Model control. **Table S2.** The differential gene changes in Model control vs FZHY treatment. **Table S3.** The unique and co-expressed genes in Normal control vs Model control vs FZHY treatment. **Table S4.** The molecular function category analysis of the differential genes. **Table S5.** The cellular component category analysis of the differential genes. **Table S6.** The biological process category analysis of the differential genes. **Table S7.** The pathway analysis of the differential genes using the KEGG pathway database.

## Data Availability

The datasets generated or analysed during this study are available from the corresponding author on reasonable request.
