# Supplementary material for: Cyp4a12-mediated retinol metabolism in stellate cells is the antihepatic fibrosis mechanism of the Chinese medicine Fuzheng Huayu recipe
Source: Chin Med. 2023 May 9;18:51. doi: 10.1186/s13020-023-00754-4 (PMC10170698; doi:10.1186/s13020-023-00754-4)
Supplement: Supplementary file 1 — Additional file 1: Figure S1. The chromatographic profile of FZHY extracts, flow rate: 1 mL/min). Peak No.: 1. danshensu; 2. protocatechuic aldehyde; 3. rosmarinic acid; 4. salvianolic acid B; 5. schizandrol A; 6. schizandrol B; 7. schizandrin A; 8. uridine 9. guanosine; 10. adenosine. Figure S2. HSCs were transfected with and without adenoviral vector for Cyp4a12a/b.Adenovirus was used to transfect 1 day HSCs for 48h and transduc-tion at MOI 1000 was the best results. Oil red staining in adenovirus-transfected HSCs was observed. Values represent means ± SD. Figure S3. The effects of FZHY on CD3+T cells expressed NKG2D.Gating strategy for flow cytometry analysis.Analysis of liver CD3+T-cells proportions and count.Frequency and number of NKG2D+-cells in liver CD3+T-cells were counted. Values represent means ± SD. [file 13020_2023_754_MOESM1_ESM.docx]

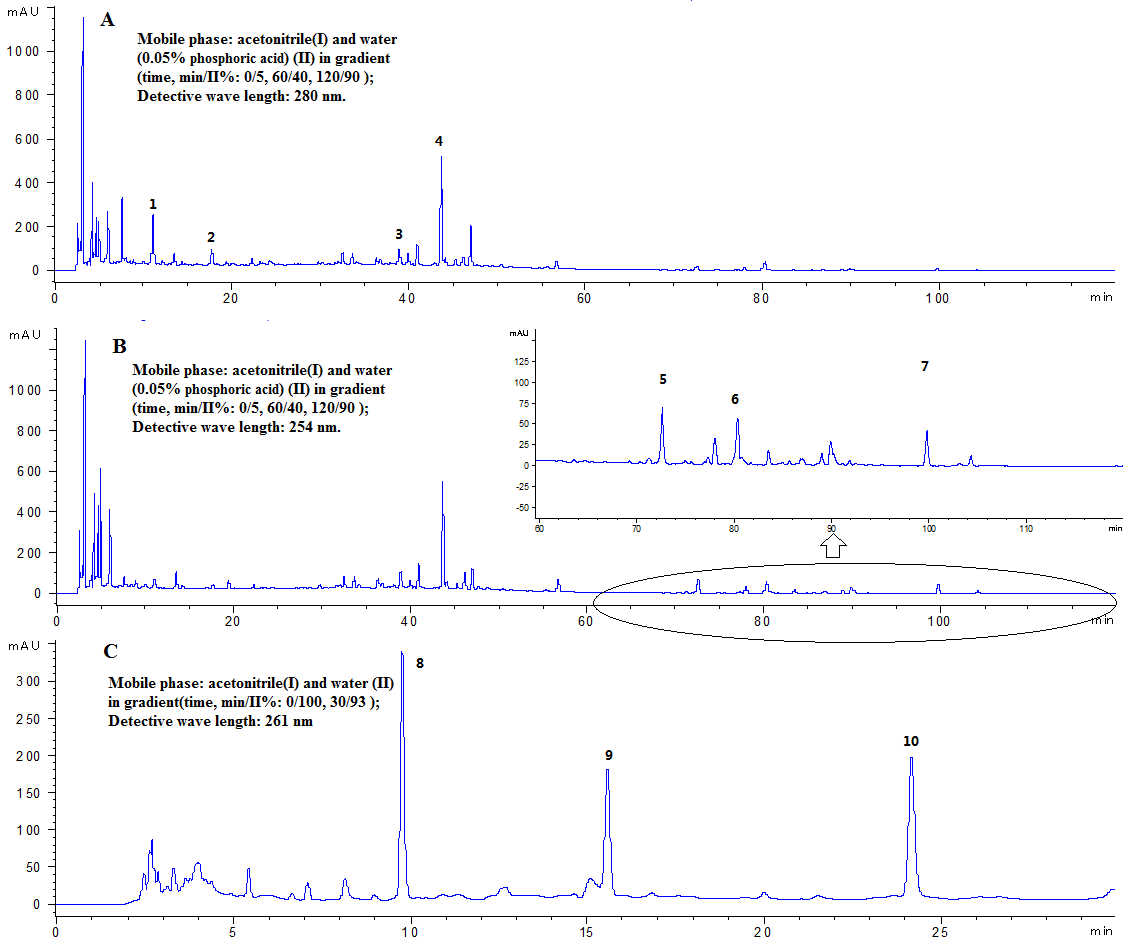


**Figure Additional 1**. The chromatographic proﬁle of FZHY extracts (Stationary phase: CNW Athena C18-WP (4.6mm×150mm, 3µm), ﬂow rate: 1 mL/min). Peak No.: 1. danshensu; 2. protocatechuic aldehyde; 3. rosmarinic acid; 4. salvianolic acid B; 5. schizandrol A; 6. schizandrol B; 7. schizandrin A; 8. uridine 9. guanosine; 10. adenosine.


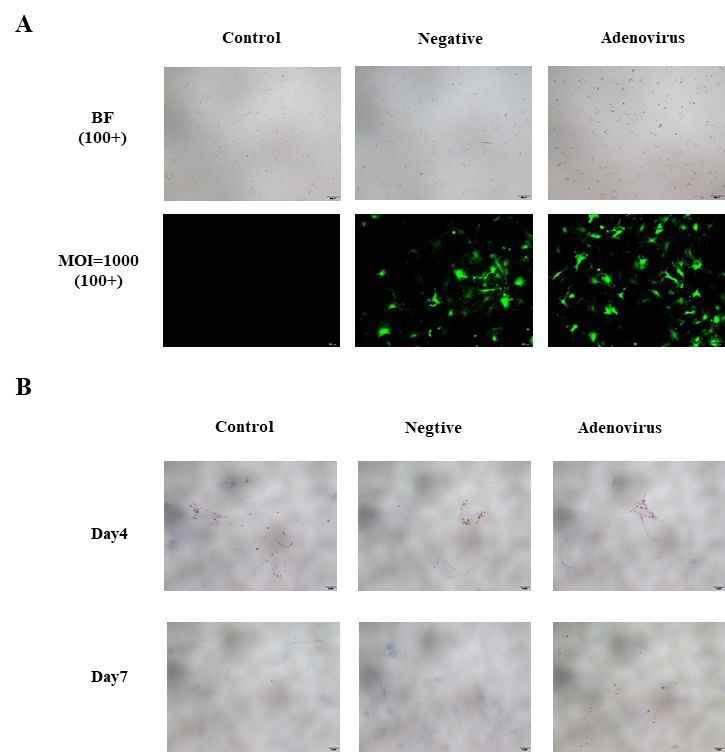


**Figure Additional 2: HSCs were transfected with and without adenoviral vector for Cyp4a12a/b.** (A) Adenovirus was used to transfect 1 day HSCs for 48h and transduc-tion at MOI 1000 was the best results. (B) Oil red staining in adenovirus-transfected HSCs was observed. Values represent means ± SD.


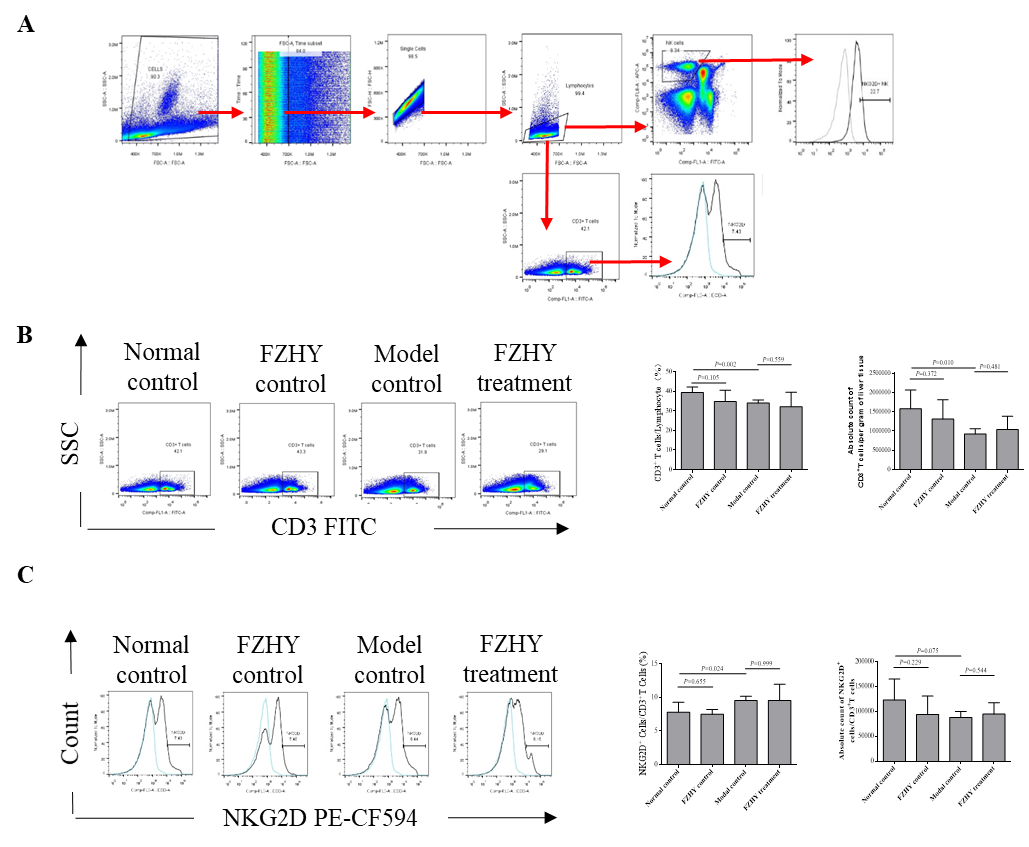


**Figure Additional 3: The effects of FZHY on CD3^+^T cells expressed NKG2D.** (A) Gating strategy for flow cytometry analysis. (B) Analysis of liver CD3^+^T-cells proportions and count. (C) Frequency and number of NKG2D^+^-cells in liver CD3^+^T-cells were counted. Values represent means ± SD.
